# Individualized duration of adjuvant alectinib therapy and rechallenge efficacy in ALK-positive non-small cell lung cancer: a case report

**DOI:** 10.3389/fmed.2026.1746932

**Published:** 2026-04-21

**Authors:** Junliang Chen, Yingni Lian, Yu Lan, Chengye Chen, Jun Chen, Zihan Zeng, Simin Feng, Meihan Wang, Tinghua Gao

**Affiliations:** 1 Department of Thoracic Oncology, The First People Hospital of Zhaoqing, Zhaoqing, Guangdong, China; 2 Department of Medical Oncology, State Key Laboratory of Oncology in South China, Sun Yat-sen University Cancer Center, Guangzhou, Guangdong, China

**Keywords:** adjuvant therapy duration, alectinib, ALK-rearranged NSCLC, CNS metastasis, rechallenge efficacy

## Abstract

**Background:**

Anaplastic lymphoma kinase (ALK)-rearranged non-small cell lung cancer (NSCLC) is a distinct molecular subtype of cancer that typically affects younger patients and carries a high risk of central nervous system (CNS) metastases. Adjuvant alectinib therapy has demonstrated efficacy in prolonging disease-free survival; however, the optimal duration of adjuvant treatment remains undefined. Moreover, the therapeutic benefit of alectinib rechallenge after disease recurrence post-adjuvant therapy remains unclear.

**Case description:**

We report the case of a 64-year-old woman diagnosed with stage pT3N2M0 IIIb ALK-positive lung adenocarcinoma. She underwent surgical resection followed by 36 months of adjuvant alectinib therapy and remained disease-free during treatment. However, 4 months after treatment discontinuation, CNS relapse occurred, manifesting as rapid neurologic progression. Reintroduction of alectinib led to marked clinical improvement and a partial radiographic response in intracranial lesions.

**Conclusion:**

This case suggests the aggressive nature of ALK-positive NSCLC and its high propensity for CNS metastasis, raising the possibility that personalized durations of adjuvant therapy guided by molecular biomarkers may be beneficial. Further research is needed to validate these approaches in larger cohorts. Furthermore, the favorable response to alectinib rechallenge observed in this case suggests that rechallenge therapy may offer a potential value in managing post-adjuvant recurrence, which warrants further investigation.

## 1 Introduction

Anaplastic lymphoma kinase (ALK)-rearranged adenocarcinoma accounts for approximately 3–8% of non-small cell lung cancer (NSCLC) cases and is characterized by distinct clinical features, including a younger age at diagnosis and a higher incidence of pleural or pericardial involvement (1). This genomic alteration results from interchromosomal inversions within the short arm of chromosome 2 [Inv(2)(p21p23)], which fuses exons 1–13 of the echinoderm microtubule-associated protein-like 4 (EML4) gene with exons 20–29 of the ALK gene. The resulting EML4-ALK fusion protein comprises an N-terminal domain derived from EML4 and a C-terminal domain that includes the intracellular tyrosine kinase segment encoded by ALK (2). The therapeutic landscape for ALK-positive NSCLC has evolved significantly with the advent of targeted therapies; most notably, the second-generation ALK tyrosine kinase inhibitor (TKI) alectinib has demonstrated robust efficacy in both advanced disease and the adjuvant setting (3, 4). The ALINA trial established 24 months of adjuvant alectinib as the current standard; however, persistent clinical heterogeneity suggests there are potential additional benefits of prolonged therapy in molecularly defined subgroups (4–6).

Second-generation ALK TKIs offer superior control of central nervous system (CNS) progression compared with first-generation agents (7). Approximately 50% of patients treated with crizotinib, a first-generation ALK inhibitor, experience CNS progression during therapy (8). In contrast, alectinib has demonstrated significant CNS protective efficacy, including an 84% reduction in the risk of CNS progression in patients with baseline CNS metastases compared with crizotinib (3). Pooled analyses have shown an intracranial objective response rate (ORR) of 42.6% (95% CI, 34.2–51.4%) in patients with baseline CNS metastases, with a median duration of CNS response of 11.1 months (95% CI, 10.3 months to not evaluable) (9).

Continuation of ALK-TKI therapy combined with localized radiotherapy remains a cornerstone in the management of oligoprogressive disease (10). Combination strategies integrating antiangiogenics agents or platinum–pemetrexed chemotherapy are typically prioritized for systemic progression (10). However, evidence regarding the effectiveness of reintroducing the original treatment regimen after progression on alectinib remains limited.

Our case analysis reveals a clinically significant pattern of rapid radiographic progression occurring shortly after the discontinuation of extended adjuvant alectinib therapy (36 months). This finding challenges current treatment paradigms and underscores the potential need for personalized adjuvant durations guided by emerging biomarkers such as molecular residual disease (MRD) monitoring. Furthermore, this report offers new perspectives on the potential clinical benefit of alectinib rechallenge following adjuvant therapy and highlights the variability in optimal treatment duration among patients. Although the findings from this case may suggest potential clinical implications, it is important to note that this report is based on a single patient. Thus, larger studies are necessary to confirm the generalizability of these observations.

## 2 Case presentation

A 64-year-old female patient with a history of thyroid cancer surgery was initially diagnosed with a primary tumor in the left lower lobe of the lung on June 1, 2021, and subsequently underwent surgical resection. Postoperative pathology confirmed stage pT3N2M0 IIIb lung adenocarcinoma. Immunohistochemical analysis of the resected specimen showed ALK (D5F3) (+), confirming ALK rearrangement positivity (Table 1). Accordingly, adjuvant therapy with alectinib (600 mg twice daily) was initiated. The patient tolerated this treatment well, with no significant adverse effects reported. Follow-up imaging performed in July 2022 showed no evidence of residual disease at the resection site. A pure ground-glass nodule in the right upper lobe was detected and subsequently monitored without intervention. Brain magnetic resonance imaging (MRI) at that time revealed no signs of CNS metastasis. The patient was informed about the current standard of care and the potential risks associated with extending treatment beyond 24 months. After discussing these factors, the patient elected to continue alectinib therapy, considering her preference for aggressive management given the high-risk disease characteristics and the favorable response to treatment. The patient continued alectinib therapy until August 2024, when treatment was discontinued. A chest computed tomography (CT) scan performed in October 2024 showed no evidence of pulmonary recurrence.

**TABLE 1:** The patient’s pathology report after radical resection of lung cancer.

Indicators	
**1. Visible to the naked eye**	
Frozen paraffin (left lower lung mass) was sent for examination. The lung mass measured 3.4 × 2.3 × 0.4 cm, was grayish-white and grayish-black, hard in texture, and showed signs of necrosis.	
The mass in the left lower lung and the gross lung tissue examined measured 16 × 9.5 × 6 cm. A mass measuring 6 × 5 × 4.5 cm was observed on the cut surface. It was grayish-white and grayish-yellow, hard in texture, and located adjacent to the pleura.	
**2. Pathological diagnosis**	
Invasive adenocarcinoma (micropapillary growth accounting for approximately 70%; acinar growth accounting for approximately 30%)	
Histological grading: moderately differentiated	
Microscopic description	
Pulmonary membrane invasion: PL0	Intra-airway dissemination: (+)
Intravascular tumor thrombus: (-)	Nerve bundle invasion: (-)
bronchial stump: (-)	
(Inferior pulmonary vein para-lymph nodes) 3 nodes, 2/3 showing adenocarcinoma metastasis.	
(Interventricular lymph nodes) 3 nodes, 1/3 showing adenocarcinoma metastasis.	
(Inferior bronchial lymph nodes) 3 nodes, 2/3 showing adenocarcinoma metastasis. CK(+).	
(Hilar lymph node) 1 node, no cancer found.	
(Inferior carinal lymph node) 1 node, showing adenocarcinoma metastasis.	
**3. Immunohistochemistry**	
p40(-), CK7(+), TTF-1(+), NapsiA(+), ***ALK(D5F3)*(+)**, ALK-N(-), PD-L1(22C3) (TPS 35%: CPS36), PD-L1(22C3-N)(-).	

Immunohistochemical staining showed ALK(D5F3)(+), confirming that the tumor was ALK-positive. Bold and italic is intended to highlight that this immunohistochemical marker indicates a diagnosis of ALK-positive NSCLC, and it has been annotated accordingly.

In December 2024, the patient was admitted to the neurosurgery department with a 10-day history of unsteady gait, left-sided limb weakness, and two seizure episodes. Brain MRI revealed multiple metastatic lesions involving the bilateral cerebral hemispheres, cerebellum, and brainstem, with a dominant lesion in the right frontal lobe exhibiting minor hemorrhage. Meningeal thickening was also noted, raising suspicion for leptomeningeal metastasis. Out of their own free will, the patient refused further treatment such as stereotactic radiosurgery (SRS), whole-brain radiotherapy, and switching to third-generation ALK inhibitors (e.g., lorlatinib). Instead, the patient resumed alectinib (600 mg twice daily) on December 14, 2024, without consultation with relevant medical professionals. Within one week, she demonstrated marked clinical improvement, including regained assisted ambulation, increased strength in the left limbs, and complete cessation of seizures.

**FIGURE 1:**
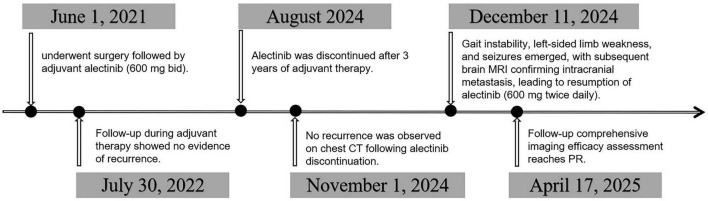
Overall treatment process.

**FIGURE 2:**
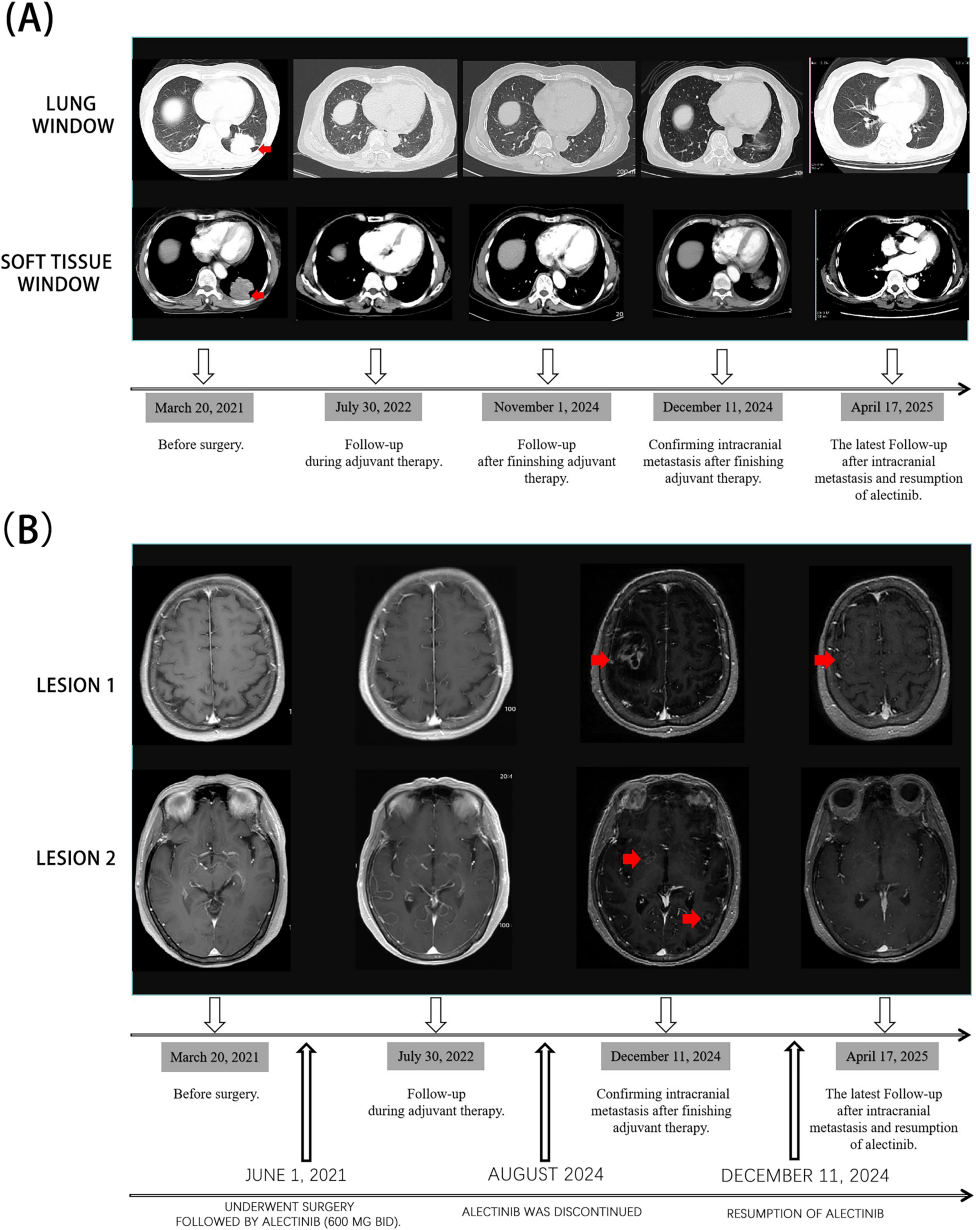
Changes in tumor burden during the patient’s treatment. **(A)** Lung and soft-tissue windows on contrast-enhanced chest CT during treatment. **(B)** Brain contrast-enhanced T1-weighted MRI during treatment.

**FIGURE 3:**
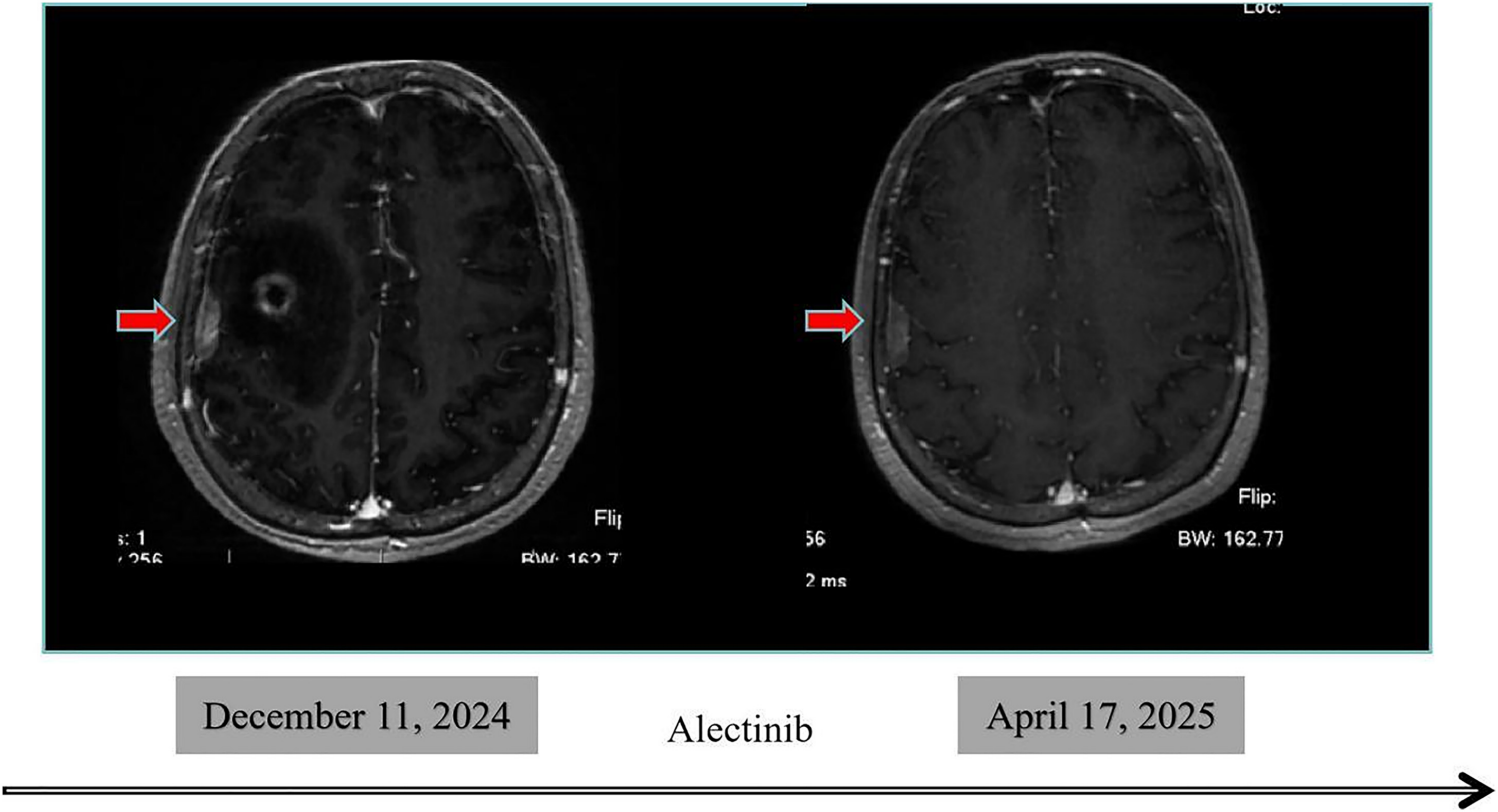
Follow-up of leptomeningeal lesions.

By April 2025, follow-up imaging revealed a partial response (PR) of the intracranial lesions according to RECIST version 1.1 criteria, with a substantial reduction in brain metastases. Chest CT continued to show no evidence of pulmonary recurrence. Physical examination during this period revealed no abnormalities in superficial lymph nodes, lung auscultation, or abdominal palpation. According to the RNAO 2.0 efficacy assessment criteria, the overall treatment efficacy was classified as PR (Table 2). The overall treatment timeline is illustrated in Figure 1, and changes in tumor burden during the course of therapy are depicted in Figure 2. The leptomeningeal lesions remained stable during alectinib treatment (Figure 3). Written informed consent was obtained from the patient for the acquisition and publication of the above clinical data and images. This manuscript presents a single-patient case report with associated clinical imaging and outcomes. The case report was conducted in accordance with the institution’s ethical standards and was granted an exemption from review under the institution’s guidelines.

**TABLE 2:** RANO 2.0 effect evaluation.

Indicators			
		December 11, 2024	April 17, 2025
Measurable lesions	Lesion 1	3.4 cm × 3.1 cm	1.6 cm × 1.5 cm
	Lesion 2	0.8 cm × 0.8 cm	Imaging invisible
New lesions		-	No
Cortisol		Not used	Not used
Clinical symptoms		Weakness in the left limbs and generalized convulsions	Significant improvement
RANO 2.0 effect evaluation		-	PR

Rechallenge therapy with alectinib in this case resulted in clinical improvement and a partial radiographic response in CNS lesions. However, due to the absence of rebiopsy and molecular analysis of resistance mechanisms, it remains uncertain whether the observed response reflects true drug sensitivity or a pharmacologic withdrawal effect, which can sometimes mimic a treatment response after drug cessation.

## 3 Discussion

Patients with ALK-positive NSCLC exhibit distinct clinical and pathological characteristics. This population predominantly includes younger patients with minimal or no smoking history (11, 12). Adenocarcinoma is the most common histological subtype, particularly the mucinous and solid variants with signet-ring cell components, in which ALK rearrangements have been detected in up to 46.2% of cases (13, 14). These tumors often demonstrate aggressive biological behavior, frequently manifesting as large primary lesions with a high incidence of nodal involvement and advanced-stage disease at diagnosis. Postoperative recurrence risk increases markedly with disease stage (5-year recurrence rates range from approximately 45% for stage IB disease to 76% for stage III disease), with the CNS being a frequent site of relapse. Conventional platinum-based adjuvant chemotherapy provides only a marginal survival benefit in this population, with an estimated improvement of approximately 5%. However, the ALINA trial demonstrated that adjuvant alectinib can significantly improve outcomes, achieving a 3-year disease-free survival (DFS) rate of 88.3% in patients with stage II–IIIA disease and reducing the risk of recurrence or death by 76% (4). Optimal management of ALK-positive NSCLC therefore requires individualized therapeutic and surveillance strategies that incorporate disease stage and molecular profiling, including the tailored use of targeted therapies, regular imaging, and CNS monitoring to optimize long-term outcomes. This present case exemplifies the aggressive clinical course and strong CNS tropism of ALK-positive NSCLC: the patient developed rapid CNS progression within 2 months after discontinuing alectinib following 3 years of adjuvant therapy. Our findings support the need for intensive, personalized post-resection follow-up and therapeutic planning in both early-stage and advanced ALK-positive NSCLC.

The ALINA trial, the first global phase III study to investigate adjuvant alectinib in patients with early-stage ALK-positive NSCLC, prespecified a treatment duration of 24 months (4). However, the optimal duration of adjuvant therapy remains a subject of ongoing clinical debate. The rationale for the 24-month regimen was largely extrapolated from prior experience with EGFR TKIs in trials such as ADAURA, as well as other targeted therapy studies (15, 16). Several factors influenced the selection of the 24-month duration: (1) increased TKI sensitivity in ALK-positive tumors, which may allow for deep remission with shorter therapy; (2) the potential to reduce the emergence of acquired resistance; (3) improved patient adherence by limiting long-term treatment burden; and (4) facilitation of regulatory approval through a standardized treatment timeframe. Despite these theoretical advantages, emerging real-world experience has highlighted the limitations of the fixed 24-month approach. Some patients experience disease recurrence even after extended therapy beyond 24 months, suggesting that a uniform duration may not be adequate for all individuals (17). Notably, the ALINA trial did not include predefined subgroup analyses according to treatment duration, resulting in an important evidence gap regarding the optimal length of adjuvant therapy. This case further illustrates the potential limitations of the current 24 month treatment paradigm. Despite extending adjuvant alectinib therapy to 36 months, the patient experienced rapid CNS relapse shortly after discontinuation. Although alectinib may suppress disease progression, it does not necessarily eliminate the underlying tumor burden, which raises the hypothesis that biomarkers such as MRD could guide more individualized durations of adjuvant therapy. However, MRD was not assessed in this case, and further research is needed to validate its role in identifying patients who may benefit from extended therapy. Thus, it is questionable whether simply extending treatment duration, as in this case, is the most effective strategy for preventing relapse. More tailored approaches are needed for patients with high-risk stage IIIb N2 disease, including indefinite therapy or biomarker-guided treatment strategies rather than arbitrary extension beyond standard durations. This raises the hypothesis that some patients may benefit from longer therapy durations; however, the evidence is insufficient, and prospective studies are needed to better define the role of treatment duration in personalized care.

Optimizing the personalized duration of adjuvant therapy for ALK-positive early-stage NSCLC patients requires more robust biomarker guidance. Dynamic MRD monitoring is a promising tool currently under investigation; however, it was not incorporated into the current patient’s clinical management. Future research should focus on assessing MRD as a potential marker to guide individualized treatment duration and identify patients at high risk of recurrence who may benefit from extended therapy (18–20). Additionally, identifying predictors of recurrence in patients receiving adjuvant therapy may help stratify treatment duration according to personalized risk profiles.

The efficacy of ALK-TKI rechallenge in patients with ALK-positive NSCLC who experience recurrence after adjuvant therapy remains uncertain due to the lack of robust preclinical or clinical evidence. However, this case may have important clinical significance. The patient experienced rapid intracranial progression shortly after discontinuing alectinib following 36 months of adjuvant therapy, yet responded favorably to rechallenge with alectinib and subsequently achieved sustained remission. This effect suggests that post-adjuvant ALK-TKI rechallenge may hold therapeutic value for selected patients. In advanced ALK-positive NSCLC, resistance mechanisms to ALK-TKIs are broadly categorized as primary or acquired. Acquired resistance is further classified into ALK-dependent and ALK-independent subtypes (21). ALK-dependent resistance primarily involves kinase domain mutations that sterically hinder TKI binding, reactivating downstream signaling (22). Compared with first-generation TKIs, second-generation inhibitors are associated with a higher incidence of ALK kinase domain mutations, each with a distinct mutational spectrum. Among these, the G1202R mutation is most frequently observed, occurring in 21, 29, and 43% of patients progressing on ceritinib, alectinib, and brigatinib, respectively (23). Although emerging data support the role of ALK-TKI rechallenge in prolonging survival in advanced-stage disease (24–26), evidence remains scarce in patients previously treated with adjuvant therapy. Prolonged exposure to ALK-TKIs in the adjuvant setting may theoretically increase the risk of resistance mutations; however, this case highlights a potential clinical rationale for rechallenge, especially in the absence of effective alternative treatments. Unfortunately, rebiopsy and genomic profiling were not performed in this case due to patient-related constraints, limiting insight into the underlying resistance mechanisms.

Despite receiving 36 months of adjuvant alectinib therapy, the patient experienced rapid CNS relapse within 4 months after treatment discontinuation. This outcome suggests that although alectinib may delay disease progression, it does not fully eradicate the residual tumor cells. Alectinib demonstrates strong CNS penetration and intracranial activity (achieving a 66.7% objective response rate in CNS patients with measurable CNS metastases); however, individual pharmacokinetic differences, blood–brain barrier integrity, metabolic characteristics, and potential drug interactions may affect the actual CNS drug exposure and efficacy (27, 28). Clinical observations indicate that alectinib can reduce cerebrovascular injury and help protect blood–brain barrier function; however, interindividual differences in barrier integrity may still limit the effective drug concentration within the brain parenchyma or meninges. Furthermore, although real-world studies indicate that even in asymptomatic and symptomatic patients with brain metastases, alectinib provides clinical benefit, some patients still experience CNS progression, suggesting that insufficient drug exposure or local microenvironmental factors may contribute to relapse (29). Treatment adherence is also crucial for maintaining effective plasma drug concentrations; and any interruption or insufficient dosage may weaken CNS disease control (30). Notably, the lack of rebiopsy and genomic analysis limits the determination of specific resistance mechanisms (e.g., secondary ALK mutations and bypass activation) (30). Existing research suggests that alectinib resistance may involve CCN1 pathway activation, metabolic reprograming, or adaptive tumor evolution in cells, whereas lipid metabolism abnormalities (such as hypercholesterolemia) have also been reported to be associated with an increased risk of CNS relapse (31). Future research should combine liquid biopsy approaches (such as CSF-ctDNA), pharmacokinetic monitoring, and multiomics analyses may help clarify individualized factors influencing CNS treatment efficacy and optimize treatment strategies and discontinuation decisions.

This case provides a valuable insight into the potential use of prolonged adjuvant therapy and rechallenge strategies in ALK-positive NSCLC. However, these findings should be interpreted cautiously. Prospective clinical trials and larger cohort studies are required to validate the feasibility and effectiveness of these strategies. Additionally, the lack of molecular profiling, including rebiopsy of CNS lesions and analysis of circulating tumor DNA (ctDNA) or ALK resistance mutations, limits our ability to definitively determine whether the observed response reflects true drug sensitivity or tumor flare phenomenon post-drug withdrawal. Furthermore, without these molecular data, it is difficult to distinguish between ALK-dependent and ALK-independent resistance mechanisms or to exclude alternative explanations, such as disease progression within pharmacologic sanctuary sites.

## 4 Conclusion

In summary, this case underscores the aggressive clinical course and robust CNS tropism of ALK-positive NSCLC. These findings raise the hypothesis that personalized treatment strategies, including individualized durations of adjuvant therapy, may be warranted. Although the favorable response to alectinib rechallenge is noteworthy, its role in managing post-adjuvant recurrence remains speculative. Further research is needed to assess biomarkers, such as MRD, to better guide personalized treatment plans.

## Data Availability

The original contributions presented in this study are included in the article/supplementary material, further inquiries can be directed to the corresponding author.

## References

[1] Kwak E Bang Y Camidge D Shaw A Solomon B Maki R et al. Anaplastic lymphoma kinase inhibition in non-small-cell lung cancer. *N Engl J Med.* (2010) 363:1693–703. 10.1056/NEJMoa1006448 20979469 PMC3014291

[2] Horn L Pao W. EML4-ALK: honing in on a new target in non-small-cell lung cancer. *J Clin Oncol.* (2009) 27:4232–5. 10.1200/JCO.2009.23.6661 19667260 PMC6955145

[3] Hida T Nokihara H Kondo M Kim Y Azuma K Seto T et al. Alectinib versus crizotinib in patients with ALK-positive non-small-cell lung cancer (J-ALEX): an open-label, randomised phase 3 trial. *Lancet.* (2017) 390:29–39. 10.1016/S0140-6736(17)30565-2 28501140

[4] Wu Y Dziadziuszko R Ahn J Barlesi F Nishio M Lee D et al. Alectinib in resected ALK-positive non-small-cell lung cancer. *N Engl J Med.* (2024) 390:1265–76. 10.1056/NEJMoa2310532 38598794

[5] Yang S. EP07. 05-10 efficacy and safety of aumolertinib in stage IB-IIIA NSCLC after R0 resection and the correlation between efficacy and postoperative MRD status. *J Thoracic Oncol.* (2023) 18:S562. 10.1016/j.jtho.2023.09.1053

[6] Chen D Guo J Huang H Tian L Xie Y Wu Q. Prognostic value of circulating tumor DNA in operable non-small cell lung cancer: a systematic review and reconstructed individual patient-data based meta-analysis. *BMC Med.* (2023) 21:467. 10.1186/s12916-023-03181-2 38012727 PMC10683311

[7] Wu L Zou Z Li Y Hao X Ying J Li J et al. Progression patterns, resistant mechanisms and subsequent therapy for ALK-positive NSCLC in the era of second-generation ALK-TKIs. *J Transl Med.* (2024) 22:585. 10.1186/s12967-024-05388-0 38902768 PMC11191366

[8] Gadgeel S Peters S Mok T Shaw A Kim D Ou S et al. Alectinib versus crizotinib in treatment-naive anaplastic lymphoma kinase-positive (ALK+) non-small-cell lung cancer: cns efficacy results from the ALEX study. *Ann Oncol.* (2018) 29:2214–22. 10.1093/annonc/mdy405 30215676 PMC6290889

[9] Gadgeel S Shaw A Govindan R Gandhi L Socinski M Camidge D et al. Pooled analysis of CNS response to alectinib in two studies of pretreated patients With ALK-positive non-small-cell lung cancer. *J Clin Oncol.* (2016) 34:4079–85. 10.1200/JCO.2016.68.4639 27863201 PMC7845943

[10] Recondo G Friboulet L. Chapter 7 - Therapeutic strategies to overcome ALK resistance in lung cancer. In: Friboulet L editor. *Therapeutic Strategies to Overcome ALK Resistance in Cancer.* Cambridge, MA: Academic Press (2021). p. 123–39.

[11] Fukui T Yatabe Y Kobayashi Y Tomizawa K Ito S Hatooka S et al. Clinicoradiologic characteristics of patients with lung adenocarcinoma harboring EML4-ALK fusion oncogene. *Lung Cancer.* (2012) 77:319–25. 10.1016/j.lungcan.2012.03.013 22483782

[12] Zhang B Zeng J Zhang H Zhu S Wang H He J et al. Characteristics of the immune microenvironment and their clinical significance in non-small cell lung cancer patients with ALK-rearranged mutation. *Front Immunol.* (2022) 13:974581. 10.3389/fimmu.2022.974581 36159860 PMC9494286

[13] Rodig S Mino-Kenudson M Dacic S Yeap B Shaw A Barletta J et al. Unique clinicopathologic features characterize ALK-rearranged lung adenocarcinoma in the western population. *Clin Cancer Res.* (2009) 15:5216–23. 10.1158/1078-0432.CCR-09-0802 19671850 PMC2865649

[14] Camidge D Kono S Flacco A Tan A Doebele R Zhou Q et al. Optimizing the detection of lung cancer patients harboring anaplastic lymphoma kinase (ALK) gene rearrangements potentially suitable for ALK inhibitor treatment. *Clin Cancer Res.* (2010) 16:5581–90. 10.1158/1078-0432.CCR-10-0851 21062932 PMC3395226

[15] Herbst R Wu Y John T Grohe C Majem M Wang J et al. Adjuvant osimertinib for resected EGFR-mutated stage IB-IIIA non-small-cell lung cancer: updated results from the phase III randomized ADAURA trial. *J Clin Oncol.* (2023) 41:1830–40. 10.1200/JCO.22.02186 36720083 PMC10082285

[16] Cheng Y Zhang X Wu L et al. Abstract CT126: Aumolertinib as adjuvant therapy in patients with stage II-IIIB EGFR-mutated NSCLC after complete tumor resection: a randomized, double-blind, placebo-controlled, phase 3 trial (ARTS). *Cancer Res.* (2025) 85:CT126–CT. 10.1158/1538-7445.AM2025-CT126 36230740

[17] Yamamoto K Toyokawa G Kozuma Y Shoji F Yamazaki K Takeo S. ALK-positive lung cancer in a patient with recurrent brain metastases and meningeal dissemination who achieved long-term survival of more than seven years with sequential treatment of five ALK-inhibitors: a case report. *Thorac Cancer.* (2021) 12:1761–4. 10.1111/1759-7714.13962 33939293 PMC8169298

[18] John T Grohe C Goldman J. Molecular residual disease (MRD) analysis from the ADAURA trial of adjuvant (adj) osimertinib in patients (pts) with resected EGFR-mutated (EGFRm) stage IB–IIIA non-small cell lung cancer (NSCLC). *J Clin Oncol.* (2024) 42:8005. 10.1200/JCO.2024.42.16_suppl.8005

[19] Zhou Y Lin Y Rao B Feng S Teng J Cheng S et al. Investigation of the role of personalized molecular residual disease in the assessment of high-risk non-small cell lung cancer (NSCLC) post-operation. *J Clin Oncol.* (2024) 42:8027. 10.1200/JCO.2024.42.16_suppl.8027

[20] Isbell J Goldstein J Hamilton E Liu Y Eichholtz J Buonocore D et al. Ultrasensitive circulating tumor DNA (ctDNA) minimal residual disease (MRD) detection in early stage non-small cell lung cancer (NSCLC). *J Clin Oncol.* (2024) 42:8078. 10.1200/JCO.2024.42.16_suppl.8078

[21] Wang Y He J Xu M Xue Q Zhu C Liu J et al. Holistic view of ALK TKI resistance in ALK-positive anaplastic large cell lymphoma. *Front Oncol.* (2022) 12:815654. 10.3389/fonc.2022.815654 35211406 PMC8862178

[22] Lovly C Shaw A. Molecular pathways: resistance to kinase inhibitors and implications for therapeutic strategies. *Clin Cancer Res.* (2014) 20:2249–56. 10.1158/1078-0432.CCR-13-1610 24789032 PMC4029617

[23] Gainor J Dardaei L Yoda S Friboulet L Leshchiner I Katayama R et al. Molecular mechanisms of resistance to first- and second-generation ALK inhibitors in ALK-rearranged lung cancer. *Cancer Discov.* (2016) 6:1118–33. 10.1158/2159-8290.CD-16-0596 27432227 PMC5050111

[24] Hochmair M Weinlinger C Prosch H. Intracranial remission with brigatinib rechallenge as fifth-line ALK inhibition therapy in a lung cancer patient. *Anticancer Drugs.* (2019) 30:1058–60. 10.1097/CAD.0000000000000800 31033499

[25] Leporati R Miliziano D Beninato T Mazzeo L Manglaviti S Brambilla M et al. Response to lorlatinib rechallenge in a case of ALK-rearranged metastatic NSCLC with a resistance mutation to second generation TKIs. *Tumori.* (2024) 110:N1–4. 10.1177/03008916241246659 38623748

[26] Browning E Weickhardt A Camidge D. Response to crizotinib rechallenge after initial progression and intervening chemotherapy in ALK lung cancer. *J Thorac Oncol.* (2013) 8:e21. 10.1097/JTO.0b013e31827a892c 23407562

[27] Wolf J Helland Å Oh IJ Migliorino MR Dziadziuszko R Wrona A et al. Final efficacy and safety data, and exploratory molecular profiling from the phase III ALUR study of alectinib versus chemotherapy in crizotinib-pretreated ALK-positive non-small-cell lung cancer. *ESMO Open.* (2022) 7:100333. 10.1016/j.esmoop.2021.100333 35042152 PMC8777286

[28] Hu Y Chang L Zhu Y Geng X Liu Z Wang R et al. Inhibition of anaplastic lymphoma kinase protects from ischemic *stroke*. *Stroke.* (2024) 55:1075–85. 10.1161/STROKEAHA.123.045991 38445502

[29] Zou Z Xing P Hao X Wang Y Song X Shan L et al. Intracranial efficacy of alectinib in ALK-positive NSCLC patients with CNS metastases-a multicenter retrospective study. *BMC Med.* (2022) 20:12. 10.1186/s12916-021-02207-x 35039026 PMC8764827

[30] Sakaguchi H Katayama R Matsumoto M Nishiyama A Matsumoto K Tajima A et al. Plasma cfDNA analysis of alectinib resistance-related gene alterations in the J-ALEX study. *ESMO Open.* (2025) 10:105574. 10.1016/j.esmoop.2025.105574 40912045 PMC12448013

[31] Bao W Tu Y Zhang S Jiang X Chen H Tu H et al. Hypercholesterolemia and the role of lipid metabolism gene CES1 in immune infiltration promote central nervous system relapse in acute myeloid leukemia. *Front Immunol.* (2025) 16:1575472. 10.3389/fimmu.2025.1575472 40771823 PMC12325197

